# Evaluating Maternal Healthcare Quality Through the Lens of Maternal near Miss: A Retrospective Analysis from a High-Volume Tertiary Center

**DOI:** 10.3390/medicina61081485

**Published:** 2025-08-19

**Authors:** İbrahim Polat, Tuğçe Arslanoğlu

**Affiliations:** 1 Department of Maternal Fetal Medicine, Basaksehir Cam and Sakura City Hospital, 34480 Istanbul, Turkey; 2 Department of Perinatology, Kanuni Sultan Süleyman Training and Research Hospital, 34307 Istanbul, Turkey; drtugcetunc@gmail.com

**Keywords:** maternal near miss, maternal mortality, life-threatening condition, mortality index, quality of healthcare

## Abstract

*Background and Objectives:* As maternal mortality has become increasingly rare in developed countries, it is no longer a reliable metric for evaluating obstetric care quality. To address this limitation, the World Health Organization (WHO) introduced the concept of maternal near miss (MNM)—a term adapted from aviation—to standardize the identification and analysis of severe maternal complications. In addition to MNM, various indices are used to assess both access to and the quality of healthcare services. *Materials and Methods:* This retrospective study evaluated all pregnant women who presented at Başakşehir Çam and Sakura City Hospital, including postpartum referrals, between May 2020 and May 2023. Given the ongoing COVID-19 pandemic during the study period, data from COVID-19-positive patients were reported separately. All definitions and classifications were based on the standardized WHO MNM criteria. *Results:* A total of 45,458 births occurred at our institution during the study period. Among the COVID-19-excluded cohort, we identified 223 life-threatening conditions (LTCs), 206 MNM cases, and 17 maternal deaths. The resulting mortality index was 7.62%. The most frequent primary diagnoses included placental invasion anomalies, severe preeclampsia, and uterine atony. The most common interventions among LTC cases were ICU admission, prolonged hospitalization, hysterectomy, and massive transfusion. *Conclusions:* Although the rates of LTCs, MNM, and maternal mortality (MM) are gradually declining, they remain essential metrics for assessing healthcare quality. This study reveals that, while tertiary centers may report higher-than-global-average indices, there remains a significant gap between current outcomes and ideal targets. Enhancing diagnostic training, optimizing intervention strategies, and implementing robust clinical algorithms are critical steps toward reducing severe maternal morbidity and mortality.

## 1. Introduction

Maternal mortality, defined as the death of a woman due to complications related to pregnancy or childbirth, continues to serve as a fundamental indicator of healthcare quality—particularly in developing countries—despite ongoing debates regarding its measurement [1]. In 2005, a population-based study by Hacettepe University revealed unexpectedly high maternal mortality rates in Turkey [2]. In response, the Turkish Ministry of Health implemented a nationwide surveillance system in 2007 that included individualized tracking of pregnant women and the deployment of emergency response teams in regions with limited healthcare access [3,4]. As a result, the maternal mortality rate was reduced from 38.3 per 100,000 live births in 2005 to 14.7 per 100,000 in 2015 [2,3,4].

Although maternal mortality remains an essential metric, its relative rarity in recent years makes it insufficient for detecting deficiencies in the quality of obstetric care. In this context, the concept of maternal near miss (MNM)—defined by the World Health Organization (WHO) as a case in which a woman nearly dies but survives a life-threatening complication during pregnancy, childbirth, or within 42 days postpartum—has emerged as a more sensitive and dynamic tool for evaluating maternal health outcomes [5].

Understanding the patterns and determinants of MNM cases at the local level can inform strategies for improving maternal healthcare, identifying high-risk groups, and implementing targeted interventions. The WHO’s standardized criteria for MNM have become a globally recognized framework for assessing severe maternal morbidity and the quality of maternity care services [5]. This study aims to determine the incidence and etiology of MNM in a high-volume tertiary center and to contribute up-to-date, context-specific data to support future improvements in maternal care practices.

## 2. Materials and Methods

All pregnant women who presented at Başakşehir Çam and Sakura City Hospital between May 2020 and May 2023 were retrospectively analyzed, regardless of whether delivery occurred at our institution. All ultrasonographic evaluations were performed via the Fujifilm Arietta 850 ultrasound system, version 1.07 (Fujifilm Healthcare, Tokyo, Japan). A convex abdominal transducer (LISENDO™ C251) was used for imaging. In addition, patients who gave birth at external facilities but who presented at our institution within the 42-day postpartum period were included.

Definitions and related indices were based on the World Health Organization (WHO) Maternal Near Miss (MNM) criteria [6], as follows: 

Maternal Near Miss (MNM): A woman who nearly died but survived a life-threatening complication during pregnancy, childbirth, or within 42 days post-termination. For clinical standardization, the presence of at least one WHO MNM criterion was required.

Life-Threatening Condition (LTC): Defined as the sum of MNM and maternal death (MD) cases (LTC = MNM + MD).

Life-Threatening Condition Ratio (LTCR): Number of LTC cases per 1000 live births (LTCR = LTC/LB).

MNM Incidence Ratio (MNM-IR): Number of MNM cases per 1000 live births, indicating the burden of severe maternal morbidity (MNM-IR = MNM/LB).

MNM to Mortality Ratio (MNM-MR): Ratio of MNM cases to maternal deaths, where higher values indicate better care quality (MNM-MR = MNM/MD).

Mortality Index (MI): Proportion of maternal deaths among LTC cases, expressed as a percentage. Higher values reflect poorer outcomes (MI = MD/LTC).

WHO MNM Criteria (selected list without footnotes):

Clinical Criteria: acute cyanosis, gasping, RR > 40 or <6/min, shock, oliguria unresponsive to treatment, clotting failure, prolonged unconsciousness, stroke, seizures, jaundice with preeclampsia, etc.

Laboratory Criteria: SpO_2_ < 90% for ≥ 60 min, pH < 7.1, PaO_2_/FiO_2_ < 200 mmHg, lactate > 5, creatinine ≥ 3.5 mg/dL, platelets < 50,000, bilirubin > 6.0 mg/dL, and others.

Management-Based Criteria: ≥5 units RBC transfusion, hysterectomy due to hemorrhage/infection, use of continuous vasoactive drugs, prolonged ventilation, dialysis, and CPR.

Laboratory-based criteria were applied only when clinically indicated. All other criteria were systematically evaluated in each patient [7]. Vaginal deliveries were observed for at least 24 h, cesarean deliveries for 48 h, and preeclampsia cases for a minimum of 72 h.

The definition of maternal death was aligned with Brain Death/Death by Neurologic Criteria (BD/DNC) [8], within the appropriate postpartum time frame. Demographic data and clinical interventions were extracted from the institutional electronic database. To prevent duplication, neonatal outcomes in multiple gestations were excluded; only maternal outcomes were assessed. Likewise, fetal data from abortions were not analyzed, while maternal outcomes were retained. Abortions were defined as pregnancies <22 weeks and <500 g; otherwise, they were classified as births.

All interventions administered to each patient were recorded, rather than selecting only the most critical types. As a result, the total number of interventions exceeded the number of patients.

### 2.1. COVID-19 Exclusion Procedure

Since the study period coincided with the COVID-19 pandemic, a sub-analysis excluding COVID-19-related cases was performed. Patients with any of the following were considered “COVID-19-suspect”:

- ICD-10 codes including U07.1, Z86.16, U09.9, Z20.822, J12.82;
- Positive COVID-19 PCR results;
- Mentions of “COVID-19” or “coronavirus” in the patient records.

All such records were manually reviewed. If COVID-19 was deemed the primary etiology of the complication, the case was excluded from the “COVID-19-excluded” analysis.

### 2.2. Statistical Analysis

All analyses were conducted using IBM SPSS Statistics Version 26 (Armonk, NY, USA). Descriptive statistics included means and standard deviations for continuous variables and frequencies and percentages for categorical variables.

### 2.3. Ethical Considerations

This study received approval from the Observational Research Ethics Committee of Başakşehir Çam and Sakura City Hospital (Reference No: 2022.06.211, approval date: 22 June 2022). The entire research process adhered to the principles of the Declaration of Helsinki [9].

## 3. Results

Between May 2020 and May 2023, a total of 45,458 live births occurred at Başakşehir Çam and Sakura City Hospital in Istanbul, Turkey. During this period, 255 patients experienced life-threatening conditions (LTCs), of which 209 were classified as maternal near miss (MNM) cases, and 46 resulted in maternal death (MD).

Given the impact of the COVID-19 pandemic on maternal outcomes, a separate analysis was conducted excluding COVID-19 cases. In this subgroup, 223 patients experienced LTCs, with 206 meeting MNM criteria and 17 resulting in maternal death.

Among the COVID-19-excluded LTCs group (*n* = 223), the mean maternal age was 31.36 ± 6.46 years (range: 16–45). The average maternal height and weight were 161.87 ± 5.28 cm and 77.53 ± 14.14 kg, respectively. The mean gestational age at the time of the event was 233.87 ± 34.37 days (range: 105–291 days), and the average neonatal birth weight was 2201.76 ± 885.16 g. The mean 1 min and 5 min Apgar scores were 5.37 ± 2.66 and 7.05 ± 2.64, respectively.

Gravidity distribution showed that 44 women (19.7%) were in their first pregnancy, 37 (16.6%) in their second, 37 (16.6%) in their third, 50 (22.4%) in their fourth, 26 (11.7%) in their fifth, 14 (6.3%) in their sixth, 7 (3.1%) in their seventh, 4 (1.8%) in their eighth, and 4 (1.8%) in their ninth.

Parity analysis revealed that 66 women (29.6%) were nulliparous, 45 (20.1%) had one prior birth, 42 (18.8%) had two, 44 (19.7%) had three, 16 (7.2%) had four, 6 (2.7%) had five, 1 (0.4%) had six, 2 (0.8%) had seven, and 1 (0.4%) had given birth eight times.

Regarding delivery mode, 200 patients (89.7%) underwent cesarean section (C-section), of whom 88 (35.6%) had a primary C-section. Among the 23 women (10.3%) who delivered vaginally, 22 (95.7%) had spontaneous vaginal deliveries, and 1 (4.3%) had an assisted vaginal delivery.

A detailed summary of the demographic characteristics and neonatal outcomes for COVID-19-excluded cases is provided in Table 1.

When calculating the relevant maternal health indicators, we included COVID-19 cases, considering them integral to maternal mortality assessment. Based on the initial figures for MNM, MD, and LTCs, the calculated indicators were as follows: the life-threatening conditions ratio (LTCR) was 4.91‰, the maternal near miss incidence ratio (MNM-IR) was 4.53‰, the maternal near miss to mortality ratio (MNM-MR) was 12.17, and the mortality index (MI) was 7.62%.

To ensure compatibility with earlier literature and to isolate the pandemic’s influence, we also calculated these indicators after excluding COVID-19 cases. A detailed comparison of both scenarios is presented in Table 2.

When the final diagnoses responsible for life-threatening conditions (LTCs) were examined, the most common etiology was placental invasion anomaly, observed in 71 patients (27.8%). This was followed by severe preeclampsia in 40 patients (15.7%), uterine atony in 32 patients (12.5%), and both eclampsia and HELLP syndrome in 20 patients each (7.8%). Less frequent causes included placental abruption, pulmonary embolism, uterine rupture, puerperal sepsis, septic abortion, ectopic pregnancy, spontaneous abortion, and cardiac complications. A detailed breakdown is provided in Table 3.

As detailed in Table 4, we present the most common medical and surgical interventions performed in patients with life-threatening conditions. Since COVID-19 cases are no longer routinely encountered in clinical practice, the primary emphasis is placed on COVID-19-excluded cases. The most frequent intervention was admission to the intensive care unit (ICU), required by 141 patients (55.29%). Additionally, 139 patients (54.51%) underwent prolonged hospitalization (>7 days), 89 patients (34.90%) required hysterectomy, 71 patients (27.84%) received massive blood transfusions (>5 units of red blood cells), 55 patients (21.01%) were administered high-dose antibiotic regimens, and 50 patients (19.61%) required mechanical ventilation. Other frequently applied interventions included the use of vasoactive drugs, relaparotomy, and dialysis. A complete breakdown of the interventions for both COVID-19-included and COVID-19-excluded cases is provided in Table 4.

## 4. Discussion

When COVID-19 cases were excluded from the analysis, the maternal near miss incidence ratio (MNM-IR) in our study was found to be 4.53 per 100 births, and the life-threatening condition ratio (LTCR) was 4.91 per 1000 live births. In Turkey, two studies conducted at different tertiary centers reported MNM-IR values of 2.47% and 5.06%, respectively [10,11]. On a global scale, the reported MNM-IR is 18.67%, while the LTCR is 6.88‰ [12,13]. Our hospital is the sixteenth largest in the world and the third largest in Turkey, functioning as a referral center for nearly all complicated obstetric cases in the region [14]. Thus, compared to other national studies, our rates fall within a reasonable range.

One international study reported average MNM-IR values ranging between 0.04–0.79% in Europe, 0.07–1.38% in North America, 0.02–5.07% in Asia, and 0.05–14.98% in Africa [15]. This comparison reveals that our center performs significantly below the levels of European and North American benchmarks. Additionally, MNM-IR and LTCR values have been shown to correlate inversely with a country’s income level [13]. In our study, while the LTCR is lower than the global average and the averages reported in low- and middle-income countries (13.44‰ and 6.35‰, respectively), it remains higher than the average for high-income countries, where LTCR is reported to be around 2.67‰ [13,16,17,18].

The high volume of near miss cases in our hospital is directly related to its status as a tertiary care center. This highlights the complexity and severity of cases referred to our institution. Despite established care protocols and experienced multidisciplinary teams, the elevated MNM indicators reflect the critical nature of our patient population. These findings reinforce the importance of a robust tiered healthcare system and underscore the need for strong clinical capacity and timely referral mechanisms at all levels of care.

Although the WHO MNM criteria were employed in this study for academic standardization, there remains no global consensus regarding their routine application [1,7,8,19]. This ongoing debate reflects the gap between the WHO-defined indicators and their practical utility in real-world clinical settings, particularly in low-resource environments where access to laboratory and interventional data may be limited. Some variables required by the WHO—such as lactate levels, PaO_2_/FiO_2_ ratios, or prolonged intubation times—are often inaccessible in such settings. Moreover, the exclusion of universally recognized life-threatening conditions, i.e., the need for intensive care unit (ICU) admission or the occurrence of eclampsia, according to the WHO’s standard criteria, further complicates implementation beyond academic use.

In this context, establishing a more universally applicable standard for MNM identification is essential to enable meaningful global comparisons of maternal care quality. Although the WHO criteria serve as a vital academic benchmark, indicators such as life-threatening conditions (LTCs) and the life-threatening condition ratio (LTCR) offer a more inclusive and practical reflection of clinical severity, particularly in facilities with high patient volume and referral complexity. Given the ongoing decline in both MNM and maternal death rates worldwide, this study emphasizes the importance of LTCs and LTCR as complementary metrics to better capture the spectrum of maternal morbidity and inform system-wide improvements.

In our study, the most frequently identified final diagnoses were placental invasion anomaly, severe preeclampsia, and uterine atony. In comparison, previous studies conducted in Turkey have reported hemorrhage, hypertensive disorders, and preeclampsia as the leading causes of maternal near miss events [10]. Larger-scale international studies have similarly identified hemorrhage, hypertensive disorders, uterine rupture, and abortion-related complications as the most common etiologies [18,20,21]. The differences observed in our cohort are likely attributable to our institution’s status as a tertiary referral center, where a higher concentration of complex cases—such as placenta accreta spectrum—are encountered. Additionally, while many global studies originate from low-resource settings, particularly in Africa, our findings reflect the patterns seen in a more resource-equipped environment.

An important methodological point is that, in contrast to other studies that classify hemorrhage by diagnosis, we categorized massive blood transfusion as an intervention rather than an etiology. This distinction may account for part of the discrepancy in cause distribution. Recognizing these key etiological factors is essential for the early identification and timely management of maternal complications. To this end, globally standardized algorithms—adaptable to both resource-rich and resource-limited settings—are urgently needed to support inexperienced practitioners and enhance the consistency of maternal care.

When analyzing the interventions implemented to prevent mortality in LTC cases, the most frequently applied procedures were ICU admission, prolonged hospitalization, hysterectomy, and massive blood transfusion. These findings are consistent with the results of previous national studies, which also reported ICU admission, massive transfusion, and hysterectomy as the most common interventions [10]. Another study employing logistic regression analysis demonstrated statistically significant associations between maternal survival and several factors, including the timing of hysterectomy, duration of hospitalization, and the volume of blood products transfused [11].

On reviewing global literature, it becomes apparent that there is a lack of standardization in the reporting and interpretation of interventional data. Despite this variability, our findings—together with national reports—underscore the critical, life-saving role of these interventions. While they are commonly included in obstetrics and gynecology training curricula, their real-world urgency and impact are often underemphasized. Therefore, increased emphasis on timely recognition and execution of these interventions in clinical training is essential to ensure effective response and optimal outcomes, particularly in high-risk scenarios.

Our study is not without limitations. The retrospective design inherently limits standardization in data recording, potentially introducing bias due to subjective interpretation—particularly in regards to demographic variables, for which some entries were incomplete. Although our hospital is among the largest tertiary centers in the country, the study remains single-centered, limiting the generalizability of findings on a global scale. Furthermore, not all laboratory-based WHO MNM criteria were uniformly tested across all patients. While necessary investigations were performed when clinically indicated, this selective testing might have led to a slight underestimation of MNM cases.

Another limitation is the absence of a control group, which precludes the establishment of statistically significant causal inferences. Despite these constraints, the present study represents the largest reported cohort from Turkey evaluating maternal near miss and mortality. We have aimed for transparency by meticulously documenting all morbidity and mortality data, and by leveraging the full spectrum of interventions available at our tertiary center. In doing so, we also sought to highlight critical educational opportunities for clinical practice improvement.

To enhance the quality and utility of future research, prospective, multicenter studies with comparative designs are essential. These studies should aim to refine and standardize MNM definitions, ensure accessibility of criteria in diverse clinical contexts, and, where appropriate, develop practical algorithms for real-world implementation.

## 5. Conclusions

Although maternal near miss (MNM), life-threatening conditions (LTCs), and maternal death (MD) rates have shown a global decline, their persistence—even in large, well-resourced tertiary centers—underscores the ongoing structural and operational challenges in maternal healthcare delivery. Our findings demonstrate that while our center exceeds global averages in terms of service capacity and intervention availability, system-level inefficiencies such as delayed referrals, fragmented perinatal care pathways, and inconsistent implementation of critical protocols likely contribute to suboptimal outcomes.

To move beyond the current plateau, actionable strategies are urgently needed. These include developing national-level triage and transfer algorithms, mandating the routine audit of MNM and LTC cases, and integrating standardized early warning systems into obstetric care. Moreover, education and simulation-based training should be intensified to promote rapid recognition and timely intervention in high-risk cases. By shifting from reactive care to anticipatory management, especially in high-volume tertiary settings, further reductions in MNM and MD rates can be achieved.

This study not only reflects the current performance of a major referral center but also highlights the need for policy-driven, system-wide reforms. Future efforts should focus on prospective, multicenter data collection using universally accepted yet contextually adaptable MNM criteria to enable valid benchmarking and drive sustainable improvements in maternal health outcomes.

## Figures and Tables

**Table 1:** Demographic variables of patients who experienced LTCs.

Variables	Mean ± SD	Min–Max (Median)
Age (Years)	31.36 ± 6.46	16–45 (32)
Height (cm)	161.87 ± 5.28	148–175 (162)
Weight (kg)	77.53 ± 14.14	51–130 (75)
Gestational Week at Birth (days)	233.87 ± 34.37	105–291 (241)
Newborn Weight (g)	2201.76 ± 885.16	250–4550 (2360)
Apgar Score at 1st min	5.37 ± 2.66	0–9 (6)
Apgar Score at 5th min	7.05 ± 2.64	0–10 (8)

Abbreviations: cm, centimeter; kg, kilogram; g, gram; min, minute.

**Table 2:** Comparative analysis of maternal health indicators, including and excluding COVID-19 cases.

Indices	Formula	Excluding COVID-19 (*n* or ‰/%)	Including COVID-19 (*n* or ‰/%)
MNM	-	206	209
MD	-	17	46
LB	-	45,458	45,458
LTC	MNM + MD	223	255
LTCR	LTC/LB	4.91‰	5.61‰
MNM-IR	MNM/LB	4.53‰	4.60‰
MNM-MR	MNM/MD	12.17	4.54
Mortality Index	MD/LTCx100	7.62%	18.04%

Abbreviations: MNM, maternal near miss; MD, maternal death; LBs, live births; LTCs, life-threatening conditions; LTCR, life-threatening condition ratio; MNM-IR, maternal near miss incidence ratio; MNM-MR, maternal near miss to mortality ratio.

**Table 3:** Distribution of final diagnoses of LTC cases (including COVID-19 cases).

Etiology	Incidence (*n*)	Percent (%)
Invasion Anomaly of the Placenta	71	27.8
Severe Preeclampsia	40	15.7
Uterine Atony	32	12.5
Eclampsia	20	7.8
HELLP Syndrome	20	7.8
Placental Abruption	16	6.3
Pulmonary Embolism	12	4.7
Uterine Rupture	10	3.9
Puerperal Sepsis	9	3.5
Septic Abortus	7	2.7
Ectopic Pregnancy	6	2.4
Abortus	6	2.4
Cardiac Complications	6	2.4

**Table 4:** Interventions in LTC and MNM cases.

	COVID-19 Cases Excluded				COVID-19 Cases Not Excluded			
	LTC (N = 223)		MNM (N = 206)		LTC (N = 255)		MNM (N = 209)	
Morbidity	Incidence (*n*)	Percent (%)	Incidence (*n*)	Percent (%)	Incidence (*n*)	Percent (%)	Incidence (*n*)	Percent (%)
ICU Administration	141	55.29	128	50.20	171	67.06	131	62.68
Hospitalization > 7 days	139	54.51	127	49.80	167	65.49	130	62.20
Hysterectomy	89	34.90	87	34.12	91	35.69	88	42.11
Massive Blood Transfusion (>5U RBCs)	71	27.84	63	24.71	79	30.98	63	30.14
High Dose Antibiotic Regimen	55	21.57	44	17.25	81	31.76	45	21.53
Mechanical Ventilation	50	19.61	35	13.73	79	30.98	36	17.22
Vasoactive Drug Administration	25	9.80	11	4.31	45	17.65	11	5.26
Relaparotomy	13	5.10	11	4.31	14	5.49	11	5.26
Dialysis	13	5.10	8	3.14	17	6.67	10	3.83

## Data Availability

Data are not publicly available due to the institutional privacy regulations of Başakşehir Çam and Sakura City Hospital but may be provided by the corresponding author upon reasonable request and ethics committee approval.

## Abbreviations

The following abbreviations are used in this manuscript:

BD/DNC	Brain Death/Death by Neurologic Criteria
C-section	Cesarean Section
LB	Live Birth
LTC	Life-Threatening Condition
LTCR	Life-Threatening Condition Ratio
min	Minute
MD	Maternal Death
MI	Mortality Index
MNM	Maternal Near Miss
MNM-IR	Maternal Near Miss Incidence Ratio
MNM-MR	Maternal Near Miss Mortality Ratio
ICU	Intensive Care Unit
RBC	Packed Red Blood Cells
WHO	World Health Organization
WMA	World Medical Association
